# 30 Years of Change: Declining Motor Fitness and Anthropometric Shifts in Polish University Students (1994–2024)

**DOI:** 10.3390/life14101325

**Published:** 2024-10-18

**Authors:** Robert Podstawski, Krzysztof Borysławski

**Affiliations:** 1 Human Wellness Research Laboratory, Department of Physiotherapy, School of Public Health, University of Warmia and Mazury in Olsztyn, 10-957 Olsztyn, Poland; 2 Institute of Health, Angelus Silesius University of Applied Sciences in Wałbrzych, 58-300 Wałbrzych, Poland; kboryslawski@ans.edu.pl

**Keywords:** motor abilities, anthropometric traits, socioeconomic status, changes over time

## Abstract

Background: To assess changes in the anthropometric and motor characteristics of male and female Polish university students between 1994 and 2024. Methods: The first study was conducted in 1994 on 712 female and 495 male university students aged 19–25 years (19.94 ± 1.09), and the second study was conducted in 2024 on 323 female and 339 male university students aged 19–25 years (19.92 ± 1.08). The participants’ body mass and height were measured, and the students participated in a modified version of Pilicz’s test consisting of four motor ability tests. The changes in the students’ performance over time were also analyzed in the context of their socioeconomic status (SES), including the place of permanent residence and the parents’ education. Results: The students’ BMI values (as well as body mass and body height in female students) were significantly higher (*p* < 0.001) in 2024 than in 1994. The male students examined in 1994 demonstrated significantly higher strength abilities in the medicine ball forward throw test. In turn, the females studied in 1994 received significantly better scores in motor ability tests, including the zig-zag run, 1-Minute Burpee Test, and the medicine ball forward throw (29.4 s, 22 cycles, 591.3 cm, respectively) than those examined in 2024 (30.1 s, 19.3 cycles, and 463.3 cm, respectively). The variations in the results were similar when the participants’ SES was considered in the analysis, which suggests that these factors had no significant effect on the analyzed characteristics over time. Conclusions: This study revealed a greater decline in the anthropometric and motor characteristics of female than male university students over a period of 30 years. The observed changes were not influenced by SES factors such as the place of permanent residence or the parents’ education.

## 1. Introduction

The university is a different environment than high school, and it is characterized by different demands and expectations. Most students successfully make the transition, but the process is not equally smooth for all participants, many of whom experience bumps in the road. Global research has long focused on university students to understand the factors affecting their health and development [1,2,3,4] for many years, and the results improve our understanding of the factors that influence the health status of the young generation. Most universities around the world have not been able to develop and implement health education programs for academic communities, which exacerbates the poor lifestyle choices made by university/college students (UCS) [5]. University students frequently develop new unhealthy practices and routines that can impact their health, lifestyle, and transition into adulthood, which is a crucial consideration because behavioral modifications are more difficult to implement in later life [6,7,8,9]. Research has shown that the transition from high school to university is a critical period for weight gain [10,11], mainly due to a poor diet [12,13] and low levels of physical activity (PA) [14,15]. In consequence, the motor fitness of UCS is also relatively low [16,17,18] and has been declining steadily in recent decades [19,20]. Physical activity is the main factor that directly influences motor fitness [21]. According to research, large proportions of university students in many countries around the world, including Finland [22], the USA [23], and the UK [24], do not meet the current PA guidelines and active lifestyle recommendations. The first study to report on the prevalence of sedentary behavior among university students was conducted nearly two decades ago [25,26,27]. Moreover, recent research has shown that universities are still settings where students spend long periods of time sitting [28,29].

Motor fitness is undoubtedly one of the key components of human health. However, motor behaviors are not an isolated function of the locomotor system, but an integral part of the human personality that evolves under continuous exposure to external factors, including those associated with the academic lifestyle [20,30,31]. University youths are an important social group in all countries around the world. Universities represent the final stage of formal learning, and they prepare selected groups of young people for mature professional and social roles. University students differ in many respects, including the place of permanent residence, social background, and aspirations.

Therefore, changes in the anthropometric and motor characteristics of university students should be explored in greater detail [32]. The changes induced by the advancement of human civilization as well as intergenerational changes can provide researchers with valuable information on the health status of successive generations [3,4,19]. Meanwhile, the vast majority of studies analyzing changes in the anthropometric and motor development of university students were conducted around three decades ago [33,34,35], and many of them investigated physical education students [2,3,4,36,37]. The results of an analysis comparing anthropometric and motor characteristics over a period of 30 years can be used to determine whether the physical and motor development of university students has improved or declined in the studied period. These findings can be used to implement appropriate corrective measures, such as physical activity programs targeting university students.

Therefore, the aim of this study was to compare the basic anthropometric (body mass, body height, and BMI) and motor (strength, speed/agility, and endurance/strength) characteristics of first-year students enrolled in the University of Warmia and Mazury in Olsztyn, Poland, in 1994 and 2024.

## 2. Materials and Methods

### 2.1. Participants

This study involved first-year students attending obligatory physical education (PE) classes in 1994 at the University of Agriculture and Technology in Olsztyn (the predecessor of the University of Warmia and Mazury in Olsztyn founded in 1999) and in 2024 at the University of Warmia and Mazury in Olsztyn (UWM), Poland. Potential participants were informed about the purpose of this study during obligatory PE, physiology, and kinesiology classes. The study conducted in 1994 involved 712 female and 495 male students aged 19–25 years (19.94 ± 1.09), whereas the study conducted in 2024 involved 323 female and 339 male students aged 19–25 years (19.92 ± 1.08). Both studies took place at the turn of April and May (spring semester), and each study lasted around three weeks. The inclusion and exclusion criteria were identical in both studies. This study involved students attending obligatory PE classes. Students participating in extracurricular PA (students engaging in sports activities on their own or as part of organized groups outside of university) were not chosen for this study because their performance could significantly affect the results. Students who were absent on the day of the evaluation or were not willing to participate were also excluded from this study. The participants were randomly selected (with the use of random number tables) from the group of students who volunteered for the research and gave their written informed consent to participate in this study. A student who was not willing to take part in this study was replaced by another randomly selected candidate. Only students who were absent on the day of the test (for whatever reason) were excluded from the random selection process. A total of 42 women and 32 men presented medical certificates or had permanent damage to locomotor organs, whereas 23 women and 17 men refused to participate without giving a reason and, therefore, did not take part in the test. This research was performed in compliance with the Declaration of Helsinki and upon the prior consent of the Bioethical Committee (No. 39/2011) of the University of Warmia and Mazury in Olsztyn.

### 2.2. Procedure, Data Collection, and Equipment

Anthropometric measurements and two motor tests (standing long jump and zig-zag run) were conducted in the first week of this study. The following two motor tests (medicine ball forward throw and 1-Min Burpee Test) were conducted in the second week of this study. Participants who were absent in the second week were tested in the third week of this study. In each week, all participants performed the first test, followed by the second test in the same order. The participants were instructed on how to correctly perform all motor tests before this study, and they were allowed time to practice. The test was preceded by an active warm-up (10 min). The warm-up routine included jogging, general and specific resistance exercises, and stretching exercises [38]. Directly before this study, the participants completed anonymous questionnaires to provide information about their sex and socioeconomic status (SES), including the place of permanent residence and their mother’s and father’s educational background. The students were asked to select one of the three provided SES options.

### 2.3. Anthropometric Measurements

Anthropometric measurements were performed immediately before the tests. Body height was measured to the nearest 0.1 mm on a WB-150 medical scale with a stadiometer (Radwag, Radom, Poland) and a Martin anthropometer Metrisis (0–2500 mm) (Metrisis GNSS, Athina, Greece) based on standard guidelines. Body mass was determined to the nearest 0.1 kg, and the results were used to calculate the participants’ BMI.

### 2.4. Assessment of Motor Fitness

Motor fitness was assessed with a modified version of Pilicz’s test battery [39] composed of four motor ability tests. The largest number of studies on the physical and motor development of Polish first-year university students was conducted by Prof. S. Pilicz between the 1960s and the 1990s [40,41,42]. For this reason, Pilicz’s test battery has been used by other Polish researchers and by the Ministry of Science and Higher Education to assess the physical development and motor fitness of Polish university students. The results of the study conducted in 1994 have never been published, which is why a similar study involving Pilicz’s test battery was undertaken 30 years later.

*The Standing Long Jump Test Which Assesses Explosive Leg Power*

Procedure: The subject stands behind the take-off line with their feet slightly apart. The subject takes off with both feet and attempts to jump forward as far as possible while swinging the arms to provide forward drive. The better score from two trials is recorded. The jump distance is measured to the nearest 1 cm from the take-off line to the back of the closest heel on landing.

Comments: The trial can be repeated if the participant crosses the take-off line before or during the jump.

The zig-zag run which assesses agility. To evaluate agility, the subject has to run a zig-zag course three times in the shortest possible time. A standard zig-zag course consists of a rectangle measuring 3 × 5 m, where cones with poles (160–180 cm in height) are placed at the corners and inside the rectangle.

Procedure: The subject stands by the start line and commences the test at the “GO” command. The athlete follows the indicated route and sprints around each cone on the left-hand side. The sprint is repeated three times without interruption. The better score from two trials is recorded.

Comments: The subject may not grab, hold onto, or move the poles. One false start is allowed. The subject is disqualified upon the second false start. The result is measured to the nearest 0.01 s using a handheld stopwatch.

The medicine ball forward throw (2 kg for women, 3 kg for men), which is a motor ability test for assessing muscle strength.

Procedure: The subject stands on a line with their feet slightly apart and holds the ball with both hands overhead. The ball is brought back behind the head and then thrown vigorously forward as far as possible without stepping over the line. The better result from two trials is recorded. The result is measured from the line to the nearest 10 cm.

Comments: The trial can be repeated if the subject steps over the line before or during the throw.

The 1-Minute Burpee Test (1-MBT), which assesses endurance/strength abilities based on the number of cycles completed in 1 min.

Procedure:

Stage I The subject begins in a standing position and moves into a supported squat with both hands on the ground.
Stage II From a supported squat, the feet are kicked back into a plank with the arms extended.
Stage III The subject returns from the plank position to a supported squat.
Stage IV The subject returns to a standing position, extends the arms over the head, and claps the hands.

The participants repeat the cycle as many times as possible within the time limit of 1 min [43].

Comments: The exercise has to be performed correctly, and the entire cycle has to be completed in the specified order. The plank position should be maintained on extended arms without arching the back, but an exception can be made for individuals without adequate upper body strength. The legs should be fully extended in the plank position. A cycle is not counted when individual stages are not correctly performed.

### 2.5. Statistical Analysis

Basic descriptive statistics (mean, SD, and range of variation) were calculated for each parameter. The normality of data distribution was verified with the Shapiro–Wilk test (skewness (As) was also examined). All tested parameters had normal distribution; therefore, the Student’s *t*-test for independent samples was used to assess the significance of the differences between the arithmetic means of the examined parameters in two cohorts. In addition, Cohen’s *d* indicator was used to assess the effect size of these differences. In sports science, Cohen’s *d* is interpreted as follows [44]: trivial (<0.2), small (0.21–0.6), moderate (0.61–1.2), large (1.21–1.99), and very large (>2.0). The differences in the socioeconomic status (SES) (place of permanent residence and parents’ education) of the students participating in both studies were also analyzed. Each SES factor was evaluated on a three-point scale (1—lowest; 3—highest) (Table 1).

The awarded points were summed up to calculate the SES score. The SES score ranged from 3 to 9 points. Based on the median value of the SES score, the participants were classified into one of two SES categories: lower SES—3–5 points; and higher SES—6–9 points.

The results were processed in the Statistica 13 program at a significance level of α = 0.05.

## 3. Results

The anthropometric and motor characteristics of male students evaluated in 1994 and 2024 are presented in Table 2. In the group of anthropometric characteristics, the BMI values of the studied males were significantly higher (*p* < 0.001) in 2024 than in 1994 (23.04 vs. 22.36 kg/m^2^). No significant differences were found in body mass and body height. In the medicine ball forward throw test, male students scored significantly lower (*p* < 0.001) results in 2024 (844.53 cm) than in 1994 (882.89 cm). The results of the remaining motor ability tests did not differ significantly between the studies (Table 2).

Much greater differences were observed in the anthropometric and motor characteristics of female students (Table 3) examined in 1994 and 2024. Body mass and BMI values were significantly higher (*p* < 0.001) in 2024 (62.2 kg and 23.6 kg/m^2^, respectively), whereas body height was significantly higher (*p* < 0.001) in 1994 (166.4 cm). In agility (the zig-zag run), endurance–strength (1-Minute Burpee Test), and strength (medicine ball forward throw) tests, female students scored significantly better (*p* < 0.001) results in 1994 (29.4 s, 22 cycles, 591.3 cm, respectively) than in 2024 (30.1 s, 19.3 cycles, and 463.3 cm, respectively). The results of the standing long jump test did not differ significantly between the studies (Table 3).

The anthropometric and motor characteristics of male and female university students divided into groups with lower and higher SES are presented in Table 4 and Table 5, respectively. In male participants with lower and higher SES, BMI values were significantly higher in 2024 (23.2, *p* < 0.001 and 22.9 kg/m^2^, *p* = 0.007, respectively). In addition, male students with higher SES scored significantly better (*p* = 0.002) results in the medicine ball forward throw test in 1994 (889.2 cm) than in 2024 (838.4 cm). No significant differences between male cohorts were noted in the remaining anthropometric and motor characteristics (Table 4).

In female participants, significant differences in anthropometric and motor characteristics were observed in the overall female population and between the two SES groups (Table 5) analyzed in 1994 and 2024. In females with lower and higher SES, the mean body mass (62.2 and 62.13 kg, respectively) and BMI (23.6 and 23.5 kg/m^2^, respectively) were significantly higher (*p* < 0.001 for all cases) in 2024. In 1994, female participants with lower and higher SES also scored significantly better (*p* < 0.001 for all cases) results in the 1-Minute Burpee Test (21.8 and 22.4 cycles per minute, respectively), medicine ball forward throw (586.3 and 599.0 cm, respectively), and the zig-zag run (only women with lower SES—29.4 s). In the remaining cases, the results scored by women from both SES groups were better in 1994 than in 2024, but the differences were not significant (*p* > 0.05, Table 5).

A different approach was used in Table 6 and Table 7, where the anthropometric and motor characteristics of male and female students from lower and higher SES groups were compared separately in each study (1994 and 2024). Male students with higher SES scored significantly better (*p* = 0.002) results in the standing long jump test in 1994 (214.8 cm) than in 2024 (208.9 cm). In turn, male participants with lower SES were significantly heavier (*p* = 0.056—significance threshold) in 2024 than in 1994 (79.0 kg vs. 77.0 kg). No significant differences (*p* < 0.05) in the remaining anthropometric and motor characteristics were noted between male students analyzed in 1994 and 2024 (Table 6).

Female participants with higher SES scored significantly better (*p* = 0.033) results in the standing long jump test in 1994 than in 2024 (214.8 vs. 208.9 cm). In 2024, women with higher SES received significantly better results in the zig-zag run (30.5 vs. 29.7 s) and significantly worse results (*p* = 0.012) in the medicine ball forward throw test (481.9 vs. 450.2 cm) relative to 1994 (Table 7).

## 4. Discussion

The present study was undertaken to compare the basic anthropometric (body mass, body height, and BMI) and motor (strength, speed/agility, and endurance/strength) characteristics of first-year university students in 1994 and 2024. The study was conducted on the assumption that the analyzed characteristics in the examined cohorts of male and female university students should change significantly over a period of 30 years.

The changes observed over 30 years were more pronounced in female students. This is a surprising outcome because many research studies [45] have demonstrated that from a biological point of view, women are characterized by greater developmental stability and are less susceptible to adverse environmental factors than men, which is manifested by the fact that women have a longer life expectancy [46,47].

In men, significant differences between 1994 and 2024 were noted only in BMI values (increase by 0.7 kg/m^2^) and strength abilities measured in the medicine ball forward throw test (decrease by 38.4 cm). These findings are not highly consistent with the results of a study conducted between 2000 and 2018 at the UWM in Olsztyn [20]. Podstawski and Żurek reported that the students evaluated in 2018 were 1.7 cm taller than those tested in 2000. Body mass and BMI values continued to decrease between 2000 and 2006 (by 0.46 kg and 0.15 kg/m^2^ per year on average), whereas a steady and significant increase in both parameters was observed between 2006 and 2018 (by 0.45 kg and 0.12 kg/m^2^ per year on average). The results of the motor tests continued to improve until 2006, after which a steady decline was observed up to 2018 when the students scored lowest in all administered motor tests [20].

A greater number of significant differences in both anthropometric and motor characteristics were observed in female participants. In 2024, the analyzed women were significantly heavier (by 4.0 kg), significantly shorter (by nearly 4.0 cm), and had a significantly higher BMI (by more than 2.5 kg/m^2^). In cross-sectional studies of female university students conducted in 2000–2018, the participants’ body mass and BMI decreased between 2000 and 2006 (by 0.24 kg and 0.18 kg/m^2^ per year on average) and increased between 2006 and 2018 (by 0.34 kg and 0.10 kg/m^2^ per year on average), whereas changes in body height followed a different trend than the remaining anthropometric characteristics. Beginning in 2000, body height increased gradually by 0.2 cm/year (0.10%), and the difference between minimum and maximum values reached 3.1 cm [19].

A greater number of significant differences in both anthropometric and motor characteristics was observed in female participants. In 2024, the analyzed women were significantly heavier (by 4.0 kg), significantly shorter (by nearly 4.0 cm), and had a significantly higher BMI (by more than 2.5 kg/m^2^). In cross-sectional studies of female university students conducted in 2000–2018, the participants’ body mass and BMI decreased between 2000 and 2006 (by 0.24 kg and 0.18 kg/m^2^ per year on average) and increased between 2006 and 2018 (by 0.34 kg and 0.10 kg/m^2^ per year on average), whereas body height increased by 3.07 cm between 2000 and 2018 [19]. In addition, Podstawski and Żurek [19,20] reported a smaller but steady increase in body height over the years, whereas in the current study, this parameter decreased significantly in women (by 3.6 cm) between 1994 and 2024. Podstawski and Żurek [19,20] also found that the results scored by both males and females were strongly correlated with their body mass and BMI. Similar correlations were observed in the authors’ previous studies examining female [48,49] and male university students [50,51]. Pribis et al. [52] reported similar correlations between motor fitness, BMI, and body fat levels of university students evaluated between 1996 and 2008. The cited study revealed a significant decline in the average fitness levels of both male and female students, measured based on their maximum oxygen uptake (VO_2 max_).

In a study of Czech physical education students [37], fluctuations (intermittent improvement and decline) were observed in the results of motor tests (anthropometric characteristics were not analyzed). In the cited research, anaerobic performance decreased in the first three years of the study (1991–1993), increased in the following eight years (1993–2001), and decreased below the initial level in 2006. A 15-year study of Hungarian university students conducted between the academic years of 1997/1998 and 2011/2012 revealed significant changes in health-related motor fitness components. The mean values of body mass, body height, BMI, and body fat percentages increased, whereas spinal flexibility and balance control declined during the examined period. In turn, the results of the handgrip test and the flexed-arm hang test were significantly better in 2011/2012 [53]. Therefore, the results described in the Hungarian study varied subject to the type of administered motor test (assessed motor ability). In some research studies evaluating secular changes in anthropometric and motor characteristics, the analyzed parameters remained fairly stable over time. Cross-sectional studies examining changes in the anthropometric and motor characteristics of physiotherapy students found them to be stable and unaffected by social factors or the fitness test, with the exception of several secular trends in the somatic features of male students (age and calf skinfold), body mass in female students, biceps skinfold in male and female students, and flexibility in females [54].

The steady increase in female students’ BMI and, consequently, the growing number of overweight women give serious cause for concern, especially in small towns and rural areas, where weight gain resulting from unhealthy lifestyle choices had been rarely reported in university students in the past [55]. According to research, around 60% of body height in adults is determined by genetic factors [56]. Therefore, around 40% of the variation in this trait is conditioned by environmental factors such as the energy balance (the relationship between food consumption and energy output, including PA) and disease burden during childhood and adolescence [57]. The cumulative and irreversible effect of environmental factors is reflected in individual height during the entire period of growth, although the impact of these factors is not equally distributed across the stages of growth [58].

In turn, the BMI is strongly determined by absolute body mass, which is regulated by different physiological mechanisms under exposure to the same environmental factors [59]. The BMI is sensitive to external stimuli and is a more labile parameter because it can both increase and decrease in response to short-term changes in the diet, PA, or health status [60].

A similar secular trend was observed when the anthropometric and motor characteristics of university students were analyzed in the context of their SES. In this case, more pronounced changes in the analyzed parameters were also noted in women. These results suggest that SES weakly influenced the examined characteristics in both males and females over time. This is a surprising outcome because previous studies investigating various populations of Polish university students demonstrated that the participants’ SES, age, and gender were responsible for differences in secular trends [19,20,30,31,61], whereas subjects from lower-income families responded most strongly to changes in environmental conditions [62]. This discrepancy could suggest that SES currently plays a less important role in the academic community and that contemporary students have similar lifestyles regardless of their place of residence and their parents’ educational attainment. At present, university students who were born in a rural area cannot be easily classified as rural residents if they spend most of their time in a city and are more influenced by the urban than the rural lifestyle. In addition to SES, rapid technological progress in the past three decades has also contributed to a decline in global fitness levels [63,64,65]. The PA levels of UCS also decreased due to changes in physical educational curricula. According to Podstawski and Sławek [66], the number of obligatory physical education classes in Polish universities decreased from 240 academic hours (45 min each) in 2000 to 60 academic hours in 2011 (which is the current standard).

Sparling and Snow [67] found that most university students with adequate PA levels were sufficiently physically active six years after graduation, while most students with insufficient PA levels remained inactive. Therefore, the results of the current study should also be analyzed in the context of the COVID-19 pandemic and lockdown measures that contributed to a sedentary lifestyle over a period of nearly three years. As a result, students’ PA levels [68,69] and motor fitness [70,71] declined regardless of environmental factors. The three-year-long pandemic was sufficiently long to reinforce the negative lifestyle habits of university students [72], and the far-reaching implications of lockdown measures eclipsed the differences in the participants’ SES.

### Strengths and Limitations

The results of cross-sectional studies comparing long-term changes in somatic and motor development are difficult to compare due to differences in the administered tests and research methods, as well as the lack of effective data distribution channels and the researchers’ reluctance to share data [73,74]. Such studies are also difficult to compare because they differ in experimental conditions, applied measurement methods, and measuring instruments [36,75,76]. Cross-sectional studies analyzing changes in anthropometric and motor characteristics should involve representative and homogeneous samples and should be conducted within the shortest time possible to avoid significant variations in experimental conditions. Therefore, the main strength of the present study was that the analyzed parameters were compared in students pursuing university degrees in the same Polish region at two points in time separated by 30 years.

The main limitation was the lack of a body composition analysis, which could not be performed in 1994 when body composition analyzers based on bioelectrical impedance were not available. In addition, Pilicz’s test battery contains relatively few motor tests, which prevented a detailed analysis of changes in specific motor abilities. However, Pilicz’s test battery was one of the most popular tests for assessing the motor fitness of Polish university students in the 1990s. Population studies involving four motor tests are also easier to organize and manage.

## 5. Conclusions

This study revealed a greater decline in the anthropometric and motor characteristics of female than male university students in the past 30 years. However, the observed changes were not strongly differentiated by factors such as the place of permanent residence or the parents’ education, which could suggest that SES factors are weakly correlated with the somatic and motor development of first-year university students. The mean BMI increased in both males and females, which may indicate that Polish first-year university students tend to gain weight. Strength abilities declined in males, whereas in females, a decline in strength, speed/agility, and endurance/strength abilities was noted in 2024 relative to 1994. Therefore, the observed changes in the somatic and motor development of Polish university students and their specific motor abilities were differentiated by sex, and these parameters should be monitored in successive years of university life.

## Figures and Tables

**Table 1:** Point scale for evaluating the participants’ socioeconomic status.

SES	Category	No. of Points
Place of permanent residence	Rural or urban area with a population of up to 10,000	1
	Urban area with a population of up to 50,000	2
	Urban area with a population higher than 50,000	3
Mother’s educational background	Primary school or secondary vocational school	1
	Secondary school of general education	2
	University degree (BA/BS, MA/MS)	3
Father’s educational background	Primary school or secondary vocational school	1
	Secondary school of general education	2
	University degree (BA/BS, MA/MS)	3

Note: SES—socioeconomic status factors.

**Table 2:** Anthropometric and motor characteristics of male students in 1994 and 2024.

Anthropometric and Motor Characteristics	1994 (n = 495)				2024 (n = 339)				Difference		
	Mean	SD	Min–Max	As	Mean	SD	Min–Max	As	*t*	*p*	Cohen’s *d*
Body mass [kg]	77.31	10.24	56.2–118.1	0.69	77.95	9.48	57.2–119.2	0.88	0.914	ns	0.065
Body height [cm]	181.47	6.07	161.4–201.6	0.511	181.68	6.39	160.2–201.8	0.09	0.476	ns	0.034
BMI [kg/m^2^]	22.36	1.75	18.26–31.65	1.98	23.04	2.62	16.08–29.66	0.40	4.543	<0.001	0.305
Standing long jump [cm]	211.97	21.03	142–271	−0.44	211.28	21.84	141–269	−0.34	−0.454	ns	0.032
Zig-zag run [s]	25.10	2.24	20.0–29.8	−1.22	25.04	2.30	20.1–30.0	−0.92	−0.038	ns	0.026
1-MBT [number of cycles]	23.36	3.51	12–29	−0.30	23.34	3.51	12–29	−0.45	−0.081	ns	0.006
Medicine ball forward throw [cm]	882.89	165.66	210–1265	−0.41	844.53	160.07	220–1286	−0.21	−3.330	<0.001	0.235

Notes: ns—not significant, 1-MBT—1-Minute Burpee Test, As—skewness, Cohen’s *d*—effect size.

**Table 3:** Anthropometric and motor characteristics of female students in 1994 and 2024.

Anthropometric and Motor Characteristics	1994 (n = 712)				2024 (n = 323)				Difference		
	Mean	SD	Min–Max	As	Mean	SD	Min–Max	As	*t*	*p*	Cohen’s *d*
Body mass [kg]	58.21	8.79	40.0–101.3	1.29	62.16	6.50	42.4–90.3	0.06	7.218	<0.001	0.511
Body height [cm]	166.37	5.82	147.1–184.3	0.06	162.78	6.70	139.8–181.4	−0.22	−8.740	<0.001	0.572
BMI [kg/m^2^]	21.03	3.02	15.02–38.22	1.88	23.57	3.10	17.05–36.78	0.39	12.407	<0.001	0.830
Standing long jump [cm]	159.03	18.99	95–210	−0.06	159.17	19.13	111–210	0.12	0.107	ns	0.007
Zig-zag run [s]	29.39	2.11	22.3–38.01	0.39	30.05	2.77	22.3–38.6	−0.06	4.197	<0.001	0.268
1-MBT [number of cycles]	22.03	3.91	7–31	0.11	19.32	4.09	9–30	−0.17	−10.180	<0.001	0.677
Medicine ball forward throw [cm]	591.28	112.33	290–1080	0.35	463.33	111.79	207–997	0.56	−17.005	<0.001	1.142

Notes: Refer to Table 2.

**Table 4:** Anthropometric and motor characteristics of male students with lower and higher SES in 1994 and 2024.

Anthropometric and Motor Characteristics	Lower SES						Higher SES					
	1994 (n = 239)		2024 (n = 161)		Difference		1994 (n = 256)		2024 (n = 178)		Difference	
	Mean	SD	Mean	SD	*t*	*p*	Mean	SD	Mean	SD	*t*	*p*
Body mass [kg]	77.58	10.61	78.98	9.90	1.32	ns	77.06	9.90	77.02	9.02	−0.04	ns
Body height [cm]	181.46	5.99	181.79	6.89	0.51	ns	181.49	6.16	181.59	5.91	0.16	ns
BMI [kg/m^2^]	22.38	1.83	23.20	2.50	3.77	<0.001	22.33	1.67	22.90	2.72	2.70	0.007
Standing long jump [cm]	208.88	21.87	211.05	21.40	0.98	ns	214.84	19.81	211.50	22.28	−1.64	ns
Zig-zag run [s]	25.24	2.21	25.08	2.30	−0.68	ns	24.97	2.26	25.01	2.30	0.21	ns
1-MBP [number of cycles]	23.32	3.65	23.44	3.61	0.34	ns	23.39	3.39	23.24	3.43	−0.46	ns
Medicine ball forward throw [cm]	876.13	160.52	851.38	150.77	−1.55	ns	889.20	170.40	838.34	168.22	−3.07	0.002

**Table 5:** Anthropometric and motor characteristics of female students with lower and higher SES in 1994 and 2024.

Anthropometric and Motor Characteristics	Lower SES						Higher SES					
	1994 (n = 403)		2024 (n = 134)		Difference		1994 (n = 309)		2024 (n = 189)		Difference	
	Mean	SD	Mean	SD	*t*	*p*	Mean	SD	Mean	SD	*t*	*p*
Body mass [kg]	58.51	9.09	62.19	7.50	4.23	<0.001	57.82	8.37	62.13	5.71	6.24	<0.001
Body height [cm]	166.01	5.52	162.62	6.66	−5.83	<0.001	166.83	6.17	162.90	6.75	−6.66	<0.001
BMI [kg/m^2^]	21.22	3.02	23.62	3.40	7.74	<0.001	20.79	3.01	23.52	2.87	10.01	<0.001
Standing long jump [cm]	158.36	18.41	156.96	18.82	−0.76	ns	159.91	19.73	160.74	19.25	0.46	ns
Zig-zag run [s]	29.43	2.12	30.53	2.86	4.76	<0.001	29.34	2.10	29.71	2.65	1.69	ns
1-MBP [number of cycles]	21.75	3.92	19.40	3.98	−5.99	<0.001	22.38	3.88	19.25	4.18	−8.48	<0.001
Medicine ball forward throw [cm]	585.33	111.35	481.85	114.517	−9.25	<0.001	599.04	113.30	450.21	108.21	−14.47	<0.001

**Table 6:** Anthropometric and motor characteristics of male students with lower and higher SES compared in each year of the study.

Anthropometric and Motor Characteristics	1994						2024					
	Lower SES (n = 239)		Higher SES (n = 259)		Difference		Lower SES (n = 161)		Higher SES (n = 178)		Difference	
	Mean	SD	Mean	SD	*t*	*p*	Mean	SD	Mean	SD	*t*	*p*
Body mass [kg]	77.58	10.61	77.06	9.90	−0.57	ns	78.98	9.8965	77.02	9.02	−1.90	0.056 *
Body height [cm]	181.46	5.99	181.49	6.16	0.07	ns	181.79	6.8890	181.59	5.91	−0.28	ns
BMI [kg/m^2^]	22.38	1.83	22.33	1.67	−0.33	ns	23.20	2.5004	22.90	2.72	−1.04	ns
Standing long jump [cm]	208.88	21.87	214.84	19.81	3.18	0.002	211.05	21.4024	211.50	22.28	0.19	ns
Zig-zag run [s]	25.24	2.21	24.97	2.26	−1.34	ns	25.08	2.3012	25.01	2.30	−0.27	ns
1-MBT [number of cycles]	23.32	3.65	23.39	3.39	0.24	ns	23.44	3.6103	23.24	3.43	−0.52	ns
Medicine ball forward throw [cm]	876.13	160.52	889.20	170.40	0.87	ns	851.38	150.77	838.34	168.22	−0.75	ns

Note: * significance threshold.

**Table 7:** Anthropometric and motor characteristics of female students with lower and higher SES compared in each year of the study.

Anthropometric and Motor Characteristics	1994						2024					
	Lower SES (n = 403)		Higher SES (n = 309)		Difference		Lower SES (n = 134)		Higher SES (n = 189)		Difference	
	Mean	SD	Mean	SD	*t*	*p*	Mean	SD	Mean	SD	*t*	*p*
Body mass [kg]	58.51	9.09	57.82	8.37	−1.03	ns	62.19	7.50	62.13	5.71	−0.08	ns
Body height [cm]	166.01	5.52	166.83	6.17	1.87	ns	162.62	6.66	162.90	6.75	0.37	ns
BMI [kg/m^2^]	21.22	3.02	20.79	3.01	−1.86	ns	23.62	3.40	23.52	2.87	−0.29	ns
Standing long jump [cm]	158.36	18.41	159.91	19.73	1.08	ns	156.96	18.82	160.74	19.25	1.76	ns
Zig-zag run [s]	29.43	2.12	29.34	2.10	−0.51	ns	30.53	2.86	29.71	2.65	−2.67	0.008
1-MBT [number of cycles]	21.75	3.92	22.38	3.88	2.14	0.033	19.40	3.98	19.25	4.18	−0.32	ns
Medicine ball forward throw [cm]	585.33	111.35	599.04	113.30	1.62	ns	481.85	114.517	450.21	108.21	−2.53	0.012

## Data Availability

Access to the Excel data generated during this study has been restricted by the Ethics Committee of the UWM in Olsztyn to protect the participants’ privacy. Researchers who meet the criteria for access to confidential data can submit a data request by email to podstawskirobert@gmail.com.
